# Supplementary figures and images for: Neighborhood level factors and use of cigarettes, cannabis and e-cigarettes: A population-based study among Canadian adults
Source: PLoS One. 2025 Nov 24;20(11):e0320035. doi: 10.1371/journal.pone.0320035 (PMC12643273; doi:10.1371/journal.pone.0320035)

Figure S1. Participant sample size for a given neighborhood measure and cigarette use

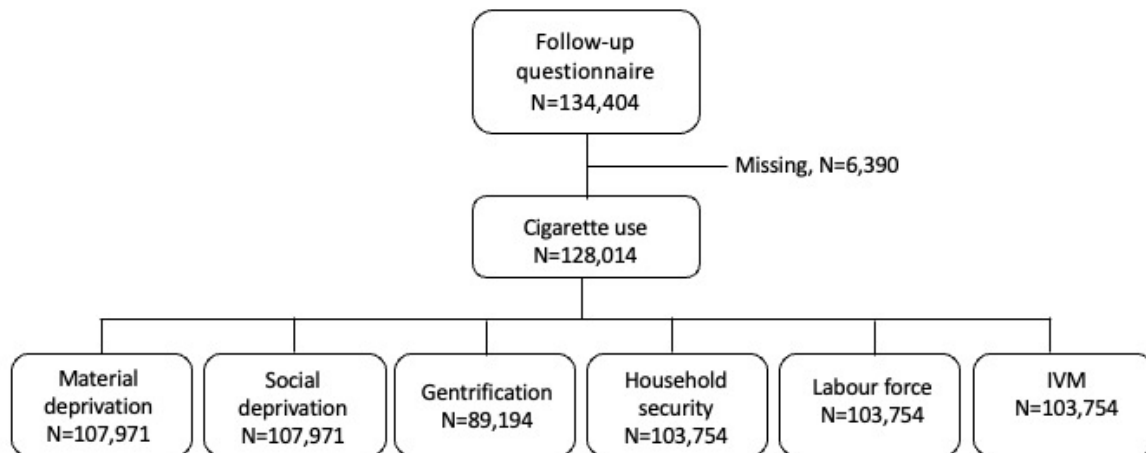

Supplement: S1 Fig — (PDF) [file pone.0320035.s001.pdf]

Figure S2. Participant sample size for a given neighborhood measure and cannabis use

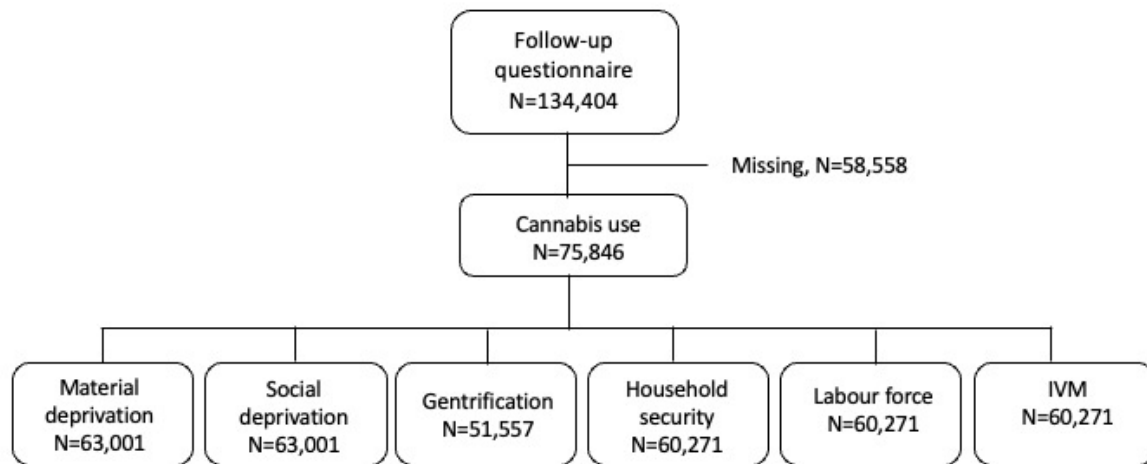

Supplement: S2 Fig — (PDF) [file pone.0320035.s002.pdf]

Figure S3. Participant sample size for a given neighborhood measure and e-cigarette use

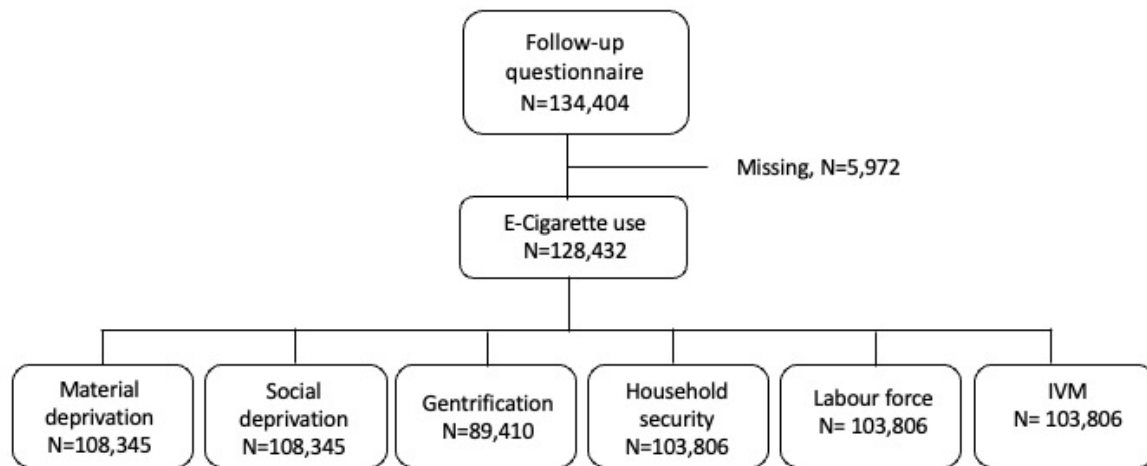

Supplement: S3 Fig — (PDF) [file pone.0320035.s003.pdf]
