# Supplementary material for: Neighborhood level factors and use of cigarettes, cannabis and e-cigarettes: A population-based study among Canadian adults
Source: PLoS One. 2025 Nov 24;20(11):e0320035. doi: 10.1371/journal.pone.0320035 (PMC12643273; doi:10.1371/journal.pone.0320035)
Supplement: S1 Table — (PDF) [file pone.0320035.s004.pdf]

S1 Table. Demographic, lifestyle, and health characteristics for participants who did not smoke (none), smoked cigarettes occasionally or daily.

Characteristic	Cigarette use		
	None<br>n = 120,669	Occasional<br>n = 1987	Daily<br>n = 5358
**Mean age (SD)**	60 (9.8)	56 (9.8)	58 (9.1)
**Female sex**	78570 (65.1%)	1239 (62.4%)	3582 (66.9%)
**Region**			
British Columbia	21572 (17.9%)	214 (10.8%)	520 (9.7%)
Alberta	28763 (23.8%)	387 (19.5%)	1230 (23.0%)
Ontario	37392 (31.0%)	721 (36.3%)	1785 (33.3%)
Quebec	12840 (10.6%)	271 (13.6%)	905 (16.9%)
Atlantic Canada	20102 (16.7%)	394 (19.8%)	918 (17.1%)
**Household income**			
< $25,000	3398 (4.5%)	83 (7.0%)	418 (12.9%)
$25,000 to 49,999	11747 (15.6%)	196 (16.6%)	794 (24.6%)
$50,000 to 74,999	15331 (20.3%)	241 (20.4%)	723 (22.4%)
$75,000 to 99,999	13765 (18.3%)	202 (17.1%)	490 (15.2%)
$99,999 to 149,999	16270 (21.6%)	244 (20.6%)	500 (15.5%)
≥$150,000	14839 (19.7%)	218 (18.4%)	305 (9.4%)
**Health perception**			
Very good to excellent	65465 (54.3%)	985 (49.6%)	2105 (39.4%)
Good	34483 (28.6%)	695 (35.0%)	2219 (41.5%)
Fair to poor	20645 (17.1%)	307 (15.5%)	1025 (19.2%)
**Marital status**			
Partnered	92092 (76.4%)	1252 (63.1%)	3068 (57.3%)
Single	28484 (23.6%)	732 (36.9%)	2282 (42.7%)
**Education**			
High school or below	18682 (16.2%)	329 (17.7%)	1518 (30.6%)
College	40862 (35.4%)	755 (40.6%)	2259 (45.6%)
Bachelors or above	55866 (48.4%)	775 (41.7%)	1177 (23.8%)
**Ethnicity**			
White	95956 (93.3%)	1567 (93.4%)	4076 (94.5%)
Non-white	6901 (6.7%)	110 (6.6%)	235 (5.5%)
**Physical activity level**			
Low	21818 (19.9%)	417 (23.8%)	1339 (29.1%)
Moderate	38704 (35.4%)	602 (34.4%)	1507 (32.7%)
High	48868 (44.7%)	732 (41.8%)	1757 (38.2%)
**BMI**			
<25.0 kg/m2	27771 (40.9%)	427 (38.2%)	1099 (39.4%)
25.0-29.9 kg/m2	23294 (34.3%)	399 (35.7%)	953 (34.1%)
≥ 30kg/m2	16792 (24.7%)	292 (26.1%)	739 (26.5%)
**Vegetables servings/day, mean (SD)**	2.9 (1.6)	2.7 (1.6)	2.2 (1.4)
**Fruit servings/day, mean (SD)**	2.3 (1.4)	2.0 (1.3)	1.6 (1.3)
**Cardiovascular disease**			
Yes	24589 (33.4%)	307 (28.7%)	911 (36.1%)

**Diabetes**			
Yes	6234 (8.5%)	112 (10.4%)	314 (12.4%)
**Mental health condition**			
Yes	15908 (23.5%)	301 (31.8%)	853 (38.9%)
**Cancer diagnosis**			
Yes	19247 (16.1%)	243 (12.4%)	792 (15.0%)

Data shown prior to imputation
