# Supplementary material for: Neighborhood level factors and use of cigarettes, cannabis and e-cigarettes: A population-based study among Canadian adults
Source: PLoS One. 2025 Nov 24;20(11):e0320035. doi: 10.1371/journal.pone.0320035 (PMC12643273; doi:10.1371/journal.pone.0320035)
Supplement: S2 Table — (PDF) [file pone.0320035.s005.pdf]

S2 Table. Demographic, lifestyle, and health characteristics for participants who used cannabis frequently, occasionally, infrequently, or not at all during the past 30 days.

Characteristic	Past 30-day cannabis use			
	None<br>n = 71377	Infrequent<br>n =1652	Occasional<br>n = 1094	Frequent<br>n = 1723
**Mean age (SD)**	61 (9.9)	56 (9.2)	56 (9.4)	56 (9.2)
**Female sex**	46419 (65.0%)	865 (52.4%)	546 (50.0%)	914 (53.0%)
**Region**				
British Columbia	11631 (16.3%)	326 (19.7%)	223 (20.4%)	339 (19.6 %)
Alberta	17544 (24.6%)	344 (20.8%)	225 (20.6%)	372 (21.6%)
Ontario	21671 (30.4%)	494 (29.9%)	335 (30.6%)	560 (32.5%)
Quebec	8532 (12.0%)	175 (10.6%)	99 (9.0%)	131 (7.6%)
Atlantic Canada	11999 (16.8%)	313 (18.9%)	212 (19.4%)	321 (18.6%)
**Household income**				
< $25,000	2303 (0.05%)	77 (0.07%)	60 (0.08%)	167 (15.0%)
$25,000 to 49,999	7826 (17.7%)	181 (16.1%)	116 (15.9%)	243 (21.9%)
$50,000 to 74,999	9595 (21.8%)	194 (17.3%)	148 (20.2%)	254 (22.8%)
$75,000 to 99,999	8059 (18.3%)	194 (17.3%)	122 (16.7%)	164 (14.7%)
$99,999 to 149,999	8802 (20.0%)	251 (22.6%)	171 (23.4%)	191 (17.2%)
≥$150,000	7520 (17.1%)	226 (20.1%)	114 (15.6%)	93 (0.08%)
**Health perception**				
Very good to excellent	38657 (54.2%)	833 (50.5%)	521 (47.8%)	648 (37.7%)
Good	21124 (29.6%)	494 (29.9%)	355 (32.5%)	648 (37.7%)
Fair to poor	11539 (16.2%)	323 (19.6%)	215 (19.7%)	424 (24.7%)
**Marital status**				
Partnered	54590 (76.6%)	1141 (69.1%)	751 (68.8%)	1044 (60.6%)
Single	16716 (23.4%)	510 (30.9%)	340 (31.2%)	679 (39.4%)
**Education**				
High school or below	12229 (17.9%)	291 (18.8%)	209 (20.3%)	405 (25.6%)
College	24612 (35.9%)	617 (39.8%)	422 (41.0%)	726 (45.9%)
Bachelors or above	31652 (46.2%)	643 (41.5%)	399 (38.7%)	452 (28.6%)
**Ethnicity**				
White	55454 (91.4%)	1329 (96.0%)	891 (95.9%)	1355 (95.9%)
Non-white	5196 (8.6%)	55 (4.0%)	38 (4.1%)	58 (4.1%)
**Physical activity level**				
Low	13470 (20.8%)	250 (17.1%)	175 (18.2%)	345 (23.7%)
Moderate	23011 (35.5%)	501 (34.3%)	298 (31.0%)	480 (33.0%)
High	28329 (43.7%)	710 (48.6%)	487 (50.7%)	4630 (3.3%)
**BMI**				
<25.0 kg/m2	15883 (39.9%)	353 (40.7%)	249 (40.4%)	357 (38.3%)
25.0-29.9 kg/m2	12811 (34.7%)	314 (36.2%)	233 (37.8%)	310 (33.3%)
≥ 30kg/m2	10133 (25.4%)	200 (23.1%)	134 (21.8%)	264 (28.4%)
**Vegetables servings/day, mean (SD)**	2.8 (1.6)	2.7 (1.6)	2.7 (1.5)	2.5 (1.7)
**Fruit servings/day, mean (SD)**	2.3 (1.4)	2.0 (1.3)	2.0 (1.3)	1.8 (1.4)
**Cardiovascular disease**				

Yes	14825 (35.3%)	250 (26.2%)	178 (28.5%)	296 (33.1%)
**Diabetes**				
Yes	3921 (9.3%)	54 (5.6%)	32 (5.1%)	85 (9.5%)
**Mental health condition**				
Yes	7972 (20.9%)	291 (33.0%)	203 (34.8%)	389 (46.6%)
**Cancer diagnosis**				
Yes	11684 (16.6%	214 (13.2%)	160 (14.8%)	253 (14.9%)

Data shown prior to imputation
