# Supplementary material for: Neighborhood level factors and use of cigarettes, cannabis and e-cigarettes: A population-based study among Canadian adults
Source: PLoS One. 2025 Nov 24;20(11):e0320035. doi: 10.1371/journal.pone.0320035 (PMC12643273; doi:10.1371/journal.pone.0320035)
Supplement: S3 Table — (PDF) [file pone.0320035.s006.pdf]

S3 Table. Demographic, lifestyle, and health characteristics for participants who reported using or not using an e-cigarette in the past 30 days.

**Characteristic**	**Past-30 day e-cigarette use**	
	No	Yes
	n = 127184	n = 1248
**Mean age (SD)**	60 (9.8)	55 (9.4)
**Female sex**	82818 (65%)	803 (64%)
**Region**		
British Columbia	22162 (17.4%)	119 (9.5%)
Alberta	30125 (23.7%)	222 (17.8%)
Ontario	39451 (31.0%)	417 (33.4%)
Quebec	14280 (11.2 %)	257 (20.6%)
Atlantic Canada	21166 (16.6%)	233 (18.7%)
**Household income**		
< $25,000	3834 (4.8%)	93 (12.2%)
$25,000 to 49,999	12701 (16.0%)	145 (19.1%)
$50,000 to 74,999	16248 (20.5%)	157 (20.6%)
$75,000 to 99,999	14421 (18.1%)	123 (16.2%)
$99,999 to 149,999	16919 (21.3%)	141 (18.5%)
≥$150,000	15302 (19.3%)	102 (13.4%)
**Health perception**		
Very good to excellent	68300 (53.7%)	504 (40.4%)
Good	37015 (29.1%)	517 (41.5%)
Fair to poor	21786 (17.1%)	226 (18.1%)
**Marital status**		
Partnered	20346 (16.8%)	298 (25.7%)
Single	43522 (35.8%)	524 (45.2%)
**Education**	57592 (47.4%)	337 (29.1%)
High school or below		
College	95930 (75.5%)	782 (62.7%)
Bachelors or above	31149 (24.5%)	466 (37.3%)
**Ethnicity**		
White	100941 (93.3%)	978 (94.7%)
Non-white	7227 (6.7%)	55 (5.3%)
**Physical activity level**		
Low	23347 (20.3%)	325 (29.9%)
Moderate	40548 (35.2%)	391 (35.9%)
High	51147 (44.5%)	372 (34.2%)
**BMI**		
<25.0 kg/m2	29064 (40.8%)	186 (38.7%)
25.0-29.9 kg/m2	24467 (34.4%)	205 (32.1%)
≥ 30kg/m2	17658 (24.8%)	247 (29.2%)
**Vegetables servings/day, mean (SD)**	2.8 (1.6)	2.3 (1.6)
**Fruit servings/day, mean (SD)**	2.2 (1.4)	1.7 (1.3)
**Cardiovascular disease**		
Yes	25630 (33.4%)	208 (33.2%)
**Diabetes**		

Yes	6597 (8.6%)	77 (12.2%)
**Mental health condition**		
Yes	16820 (24.0%)	232 (44.4%)
**Cancer diagnosis**		
Yes	20104 (16.0%)	159 (13.1%)

Data shown prior to imputation
