# Supplementary material for: Neighborhood level factors and use of cigarettes, cannabis and e-cigarettes: A population-based study among Canadian adults
Source: PLoS One. 2025 Nov 24;20(11):e0320035. doi: 10.1371/journal.pone.0320035 (PMC12643273; doi:10.1371/journal.pone.0320035)
Supplement: S4 Table — (PDF) [file pone.0320035.s007.pdf]

S4 Table. Descriptive statistics, N (%), for the prevalence of neighborhood factors within levels of cigarette use.

**Individual or neighborhood factor**	**Cigarette use**		
	None	Occasional	Daily
**Neighborhood material deprivation**			
Quintile 1	34884 (34.2%)	514 (30.6%)	953 (22.0%)
Quintile 2	24591 (24.1%)	398 (23.7%)	951 (22.0%)
Quintile 3	19115 (18.7%)	334 (19.9%)	904 (20.9%)
Quintile 4	14711 (14.4%)	237 (14.1%)	846 (19.5%)
Quintile 5	8659 (8.5%)	197 (11.7%)	677 (15.6%)
**Neighborhood social deprivation**			
Quintile 1	20561 (20.2%)	276 (16.4%)	535 (12.4%)
Quintile 2	20131 (19.7%)	275 (16.4%)	715 (16.5%)
Quintile 3	21446 (21.0%)	301 (17.9%)	862 (19.9%)
Quintile 4	20752 (20.4%)	396 (23.6%)	1026 (23.7%)
Quintile 5	19070 (18.7%)	432 (25.7%)	1193 (27.5%)
**Living in a gentrified neighborhood**			
Yes	14845 (17.6%)	336 (24.2%)	716 (20.7%)
**Neighborhood household security**			
Quintile 1	20721 (21.1%)	265 (16.6%)	554 (14.0%)
Quintile 2	21281 (21.7%)	310 (19.5%)	721 (18.2%)
Quintile 3	20697 (21.1%)	313 (19.6%)	864 (21.8%)
Quintile 4	17907 (18.2%)	310 (19.5%)	896 (22.6%)
Quintile 5	17597 (17.9%)	395 (24.8%)	923 (23.3%)
**Neighborhood labour force**			
Quintile 1	18764 (19.1%)	355 (22.3%)	788 (19.9%)
Quintile 2	20123 (20.5%)	317 (19.9%)	751 (19.0%)
Quintile 3	20114 (20.5%)	329 (20.7%)	848 (21.4%)
Quintile 4	19901 (20.3%)	298 (18.7%)	786 (19.9%)
Quintile 5	19301 (19.7%)	294 (18.5%)	785 (19.8%)
**Neighborhood IVM**			
Quintile 1	14962 (15.2%)	257 (16.1%)	739 (18.7%)
Quintile 2	19784 (20.1%)	328 (20.6%)	881 (22.3%)
Quintile 3	23969 (24.4%)	393 (24.7%)	909 (23.0%)
Quintile 4	23844 (24.3%)	375 (23.5%)	821 (20.7%)
Quintile 5	15644 (15.9%)	240 (15.1%)	608 (15.4%)
