# Supplementary material for: Neighborhood level factors and use of cigarettes, cannabis and e-cigarettes: A population-based study among Canadian adults
Source: PLoS One. 2025 Nov 24;20(11):e0320035. doi: 10.1371/journal.pone.0320035 (PMC12643273; doi:10.1371/journal.pone.0320035)
Supplement: S5 Table — (PDF) [file pone.0320035.s008.pdf]

S5 Table. Descriptive statistics, N (%), for the prevalence of neighborhood factors within levels of past 30-day cannabis use.

Neighborhood factor	Past 30-day cannabis use			
	None	Infrequent	Occasional	Frequent
**Neighborhood material deprivation**				
Quintile 1	19055 (32.2%)	471 (33.5%)	290 (31.7%)	370 (25.7%)
Quintile 2	14391 (24.3%)	343 (24.4%)	200 (21.9%)	287 (19.9%)
Quintile 3	11470 (19.4%)	243 (17.3%)	172 (18.8%)	284 (19.7%)
Quintile 4	8894 (15.0%)	211 (15.0%)	162 (17.7%)	278 (19.3%)
Quintile 5	5431 (9.2%)	136 (9.7%)	91 (9.9%)	222 (15.4%)
**Neighborhood social deprivation**				
Quintile 1	12604 (21.3%)	214 (15.2%)	128 (14.0%)	143 (9.9%)
Quintile 2	11954 (20.2%)	213 (15.2%)	137 (15.0%)	236 (16.4%)
Quintile 3	12366 (20.9%)	310 (22.1%)	196 (21.4%)	272 (18.9%)
Quintile 4	11890 (20.1%)	303 (21.6%)	212 (23.2%)	370 (25.7%)
Quintile 5	10427 (17.6%)	364 (25.9%)	242 (26.4%)	420 (29.1%)
**Living in a gentrified neighborhood**				
Yes	7656 (15.8%)	326 (28.0%)	212 (27.2%)	345 (29.8%)
**Neighborhood household security**				
Quintile 1	12570 (22.2%)	198 (14.9%)	104 (11.6%)	161 (11.5%)
Quintile 2	12494 (22.1%)	229 (17.2%)	173 (19.2%)	233 (16.6%)
Quintile 3	12000 (21.2%)	284 (21.4%)	193 (21.4%)	326 (23.2%)
Quintile 4	10102 (17.8%)	274 (20.6%)	198 (22.0%)	296 (21.1%)
Quintile 5	9470 (16.7%)	344 (25.9%)	232 (25.8%)	390 (27.7%)
**Neighborhood labour force**				
Quintile 1	10419 (18.4%)	284 (21.4%)	194 (21.6%)	282 (20.1%)
Quintile 2	11290 (19.9%)	273 (20.5%)	175 (19.4%)	310 (22.0%)
Quintile 3	11594 (20.5%)	264 (19.9%)	193 (21.4%)	275 (19.6%)
Quintile 4	11767 (20.8%)	265 (19.9%)	180 (20.0%)	256 (18.2%)
Quintile 5	11566 (20.4%)	243 (18.3%)	158 (17.6%)	283 (20.1%)
**Neighborhood IVM**				
Quintile 1	8853 (15.6%)	207 (15.6%)	166 (18.4%)	256 (18.2%)
Quintile 2	11309 (20.0%)	303 (22.8%)	175 (19.4%)	307 (21.8%)
Quintile 3	13490 (23.8%)	333 (25.1%)	234 (26.0%)	312 (22.2%)
Quintile 4	13288 (23.5%)	302 (22.7%)	209 (23.2%)	320 (22.8%)
Quintile 5	9696 (17.1%)	184 (13.8%)	116 (12.9%)	211 (15.0%)
