# Supplementary material for: Neighborhood level factors and use of cigarettes, cannabis and e-cigarettes: A population-based study among Canadian adults
Source: PLoS One. 2025 Nov 24;20(11):e0320035. doi: 10.1371/journal.pone.0320035 (PMC12643273; doi:10.1371/journal.pone.0320035)
Supplement: S6 Table — (PDF) [file pone.0320035.s009.pdf]

S6 Table. Descriptive statistics, N (%), for the prevalence of neighborhood factors among participants who did and did not use an e-cigarette in the past 30-days.

**Neighborhood factor**	**Past 30-day e-cigarette use**	
	No	Yes
**Neighborhood material deprivation**		
Quintile 1	36180 (33.7%)	265 (25.2%)
Quintile 2	25800 (24.0%)	237 (22.5%)
Quintile 3	20223 (18.8%)	223 (21.2%)
Quintile 4	15668 (14.6%)	184 (17.5%)
Quintile 5	9422 (8.8%)	143 (12.6%)
**Neighborhood social deprivation**		
Quintile 1	21302 (19.9%)	153 (14.5%)
Quintile 2	21022 (19.6%)	171 (16.3%)
Quintile 3	22472 (20.9%)	212 (20.1%)
Quintile 4	22005 (20.5%)	234 (22.2%)
Quintile 5	20492 (19.1%)	282 (26.8%)
**Living in a gentrified neighborhood**		
Yes	15758 (17.8%)	183 (20.5%)
**Neighborhood household security**		
Quintile 1	21397 (20.8%)	156 (16.4%)
Quintile 2	22118 (21.5%)	191 (20.1%)
Quintile 3	21687 (21.1%)	193 (20.3%)
Quintile 4	18938 (18.4%)	197 (20.8%)
Quintile 5	18717 (18.2%)	212 (22.3%)
**Neighborhood labour force**		
Quintile 1	19702 (19.2%)	186 (22.6%)
Quintile 2	21034 (20.4%)	186 (18.8%)
Quintile 3	21098 (20.5%)	186 (21.0%)
Quintile 4	20814 (20.2%)	186 (19.6%)
Quintile 5	20209 (19.6%)	172 (18.1%)
**Neighborhood IVM**		
Quintile 1	15808 (15.4%)	155 (16.3%)
Quintile 2	20819 (20.2%)	204 (21.5%)
Quintile 3	25022 (24.3%)	252 (26.6%)
Quintile 4	24848 (24.2%)	216 (22.8%)
Quintile 5	16360 (15.9%)	122 (12.9%)
